# Supplementary figures and images for: Adipose-derived exosomal miR-421 targets CBX7 and promotes metastatic potential in ovarian cancer cells
Source: J Ovarian Res. 2023 Nov 30;16:233. doi: 10.1186/s13048-023-01312-0 (PMC10688490; doi:10.1186/s13048-023-01312-0)

Supplementary Figure 1

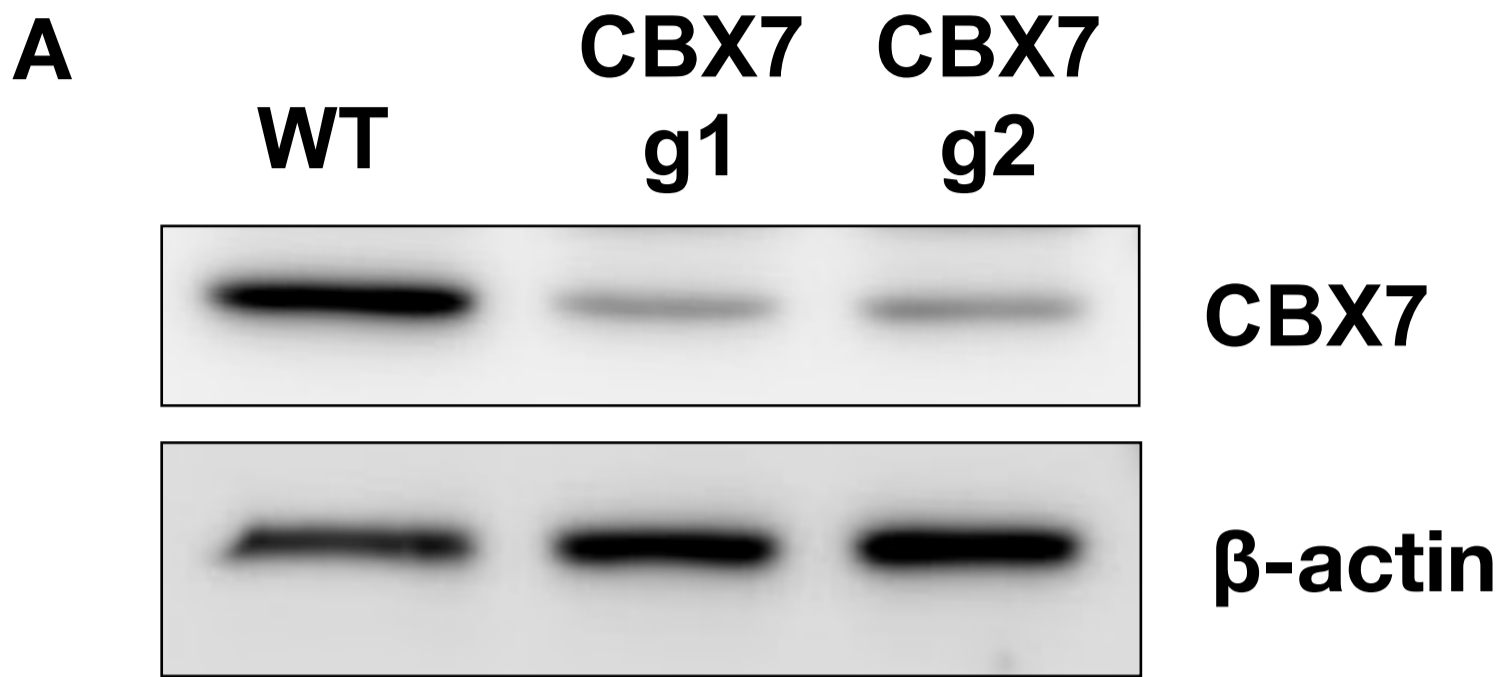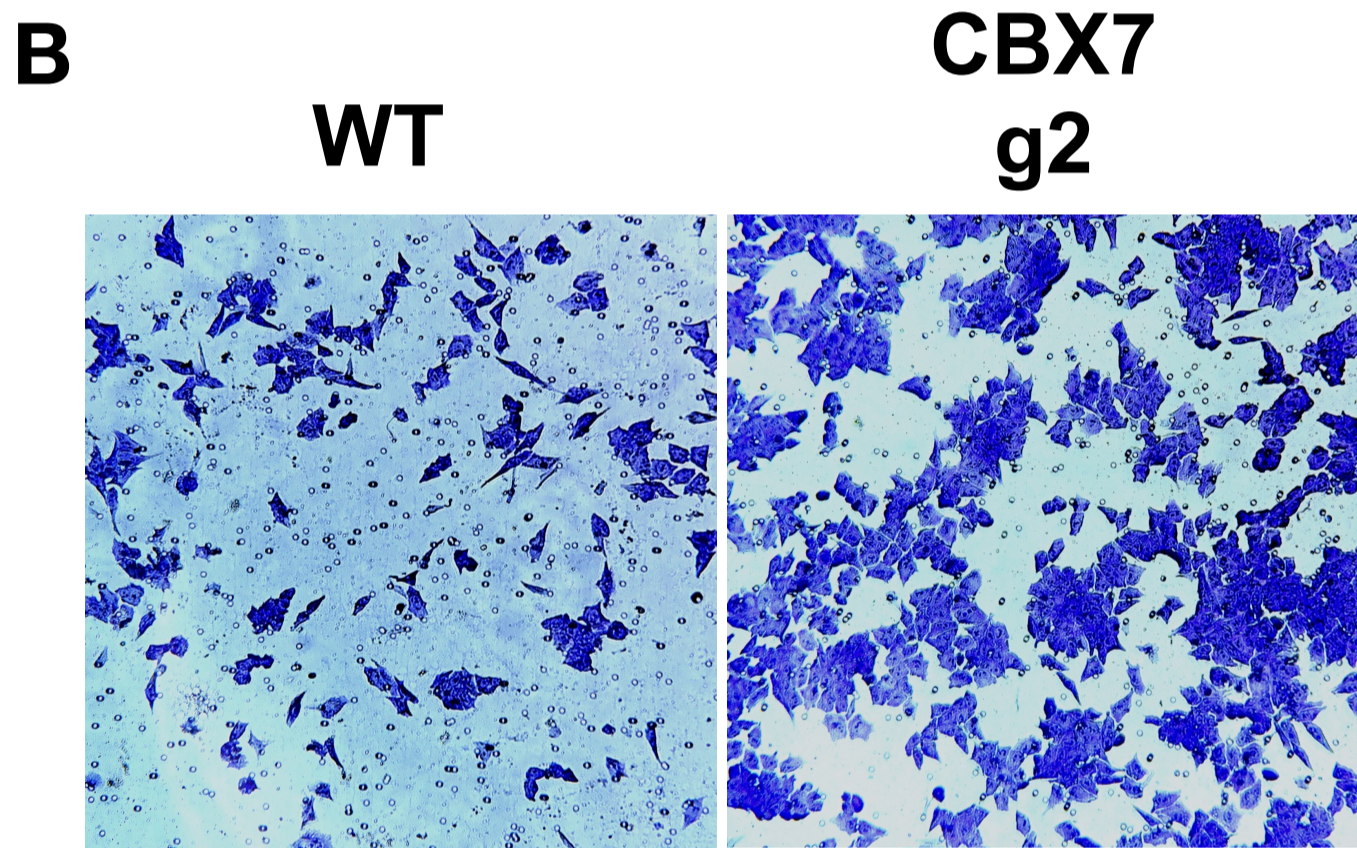

Supplementary Figure 2

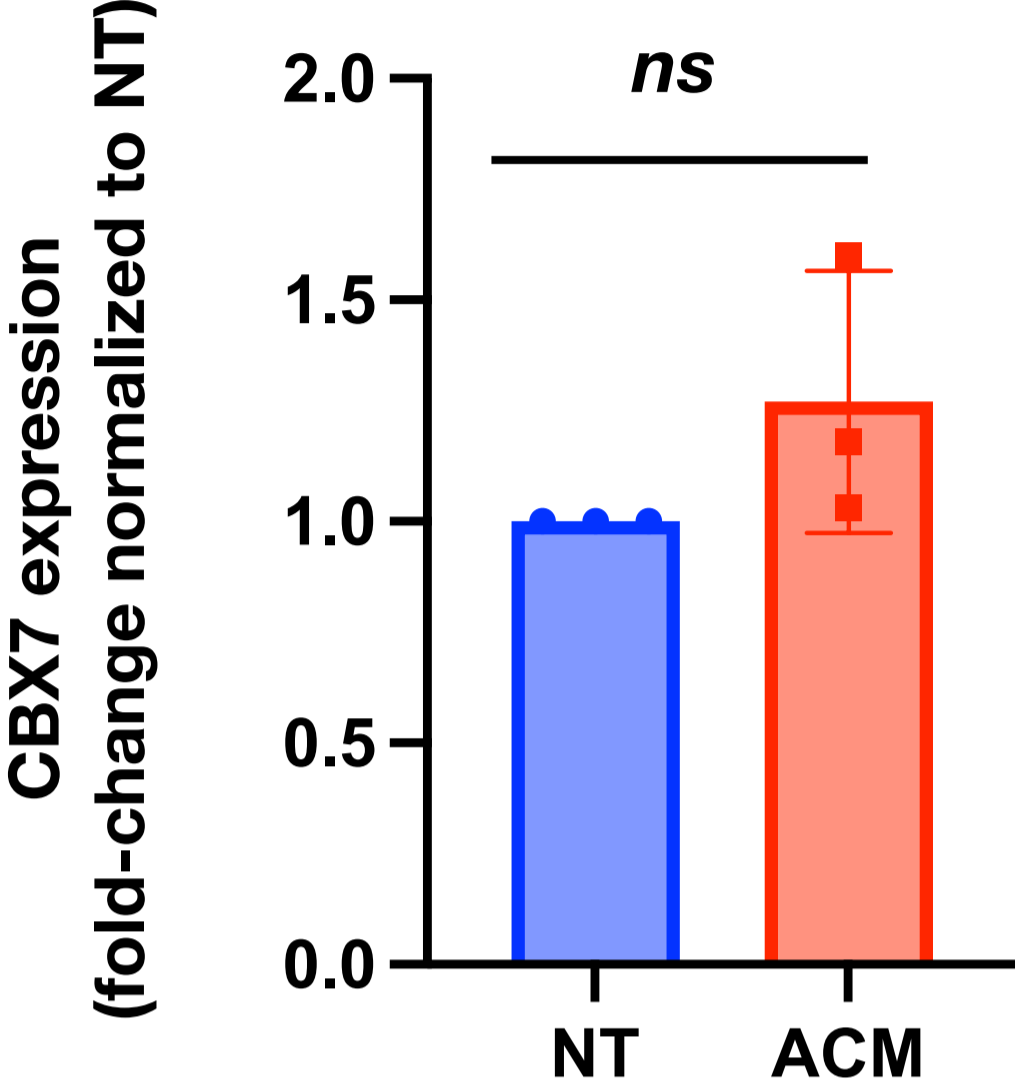

Supplement: Supplementary file 1 — Additional file 1: Supplementary Figure 1. Loss of CBX7 is sufficient to enhance migration. (A) CBX7 was stably knocked-down in OCSC1-F2 human OC cells using CRISPR-Cas9. (A) Expression of CBX7 was determined by Western blot analysis; (B) Effect on migration was determined using trans-well migration assay. WT, wild-type; g1, guide RNA 1; g2, guide RNA 2. Supplementary Figure 2. CBX7 protein downregulation is not associated with changes in mRNA. OVCAR3 and OCSC1-F1 human OC cells were cultured in ACM for 5 days and effect on CBX7 mRNA was determined by qPCR. NT, Control no treatment cells were cultured in growth media. [file 13048_2023_1312_MOESM1_ESM.pdf]
